# Adverse influence of bisoprolol on central blood pressure in the upright position: a double-blind placebo-controlled cross-over study

**DOI:** 10.1038/s41371-019-0188-9

**Published:** 2019-03-18

**Authors:** Lauri Suojanen, Antti Haring, Antti Tikkakoski, Heini Huhtala, Mika Kähönen, Arttu Eräranta, Jukka T. Mustonen, Ilkka H. Pörsti

**Affiliations:** 1 Faculty of Medicine and Health Technology, P.O. Box 100, FIN-33014 University of Tampere, Tampere, Finland; 2 Faculty of Social Sciences, P.O. Box 100, FIN-33014 University of Tampere, Tampere, Finland; 3 Department of Clinical Physiology and Nuclear Medicine, Tampere University Hospital, P.O. Box 2000, 33521 Tampere, Finland; 4 Department of Internal Medicine, Tampere University Hospital, P.O. Box 2000, 33521 Tampere, Finland

**Keywords:** Clinical trials, Hypertension, Hypertension

## Abstract

Treatment with beta-blockers is characterized by inferior reduction of central versus peripheral blood pressure. We examined changes in blood pressure, cardiac function, and vascular resistance after 3 weeks of bisoprolol treatment (5 mg/day) during passive head-up tilt in 16 never-treated Caucasian males with grade I–II primary hypertension. A double-blind, randomized, placebo-controlled cross-over design was applied, and hemodynamics were recorded using continuous tonometric pulse wave analysis and whole-body impedance cardiography. Bisoprolol decreased blood pressure in the aorta (~8/10 mmHg, *p* ≤ 0.032) and radial artery (~10/9 mmHg, *p* ≤ 0.037), but upright aortic systolic blood pressure was not significantly reduced (*p* = 0.085). Bisoprolol reduced heart rate and left cardiac work, and increased subendocardial viability index in supine and upright positions (*p* ≤ 0.044 for all). Bisoprolol increased stroke volume in the supine (~11 ml, *p* = 0.02) but not in the upright position, while only upright (~1 l/min, *p* = 0.007) but not supine cardiac output was reduced. Upright elevation in systemic vascular resistance was increased 2.7-fold (*p* = 0.002), while upright pulse pressure amplification was decreased by ~20% (*p* = 0.002) after bisoprolol. Aortic augmentation index, augmentation pressure, and pulse pressure were not changed in the supine position but were increased in the upright position (from 9% to 17%, 3–6 mmHg, and 30–34 mmHg, respectively, *p* ≤ 0.016 for all). In conclusion, although bisoprolol treatment reduced peripheral blood pressure, central systolic blood pressure in the upright position was not decreased. Importantly, the harmful influences of bisoprolol on central pulse pressure and pressure wave reflection were manifested in the upright position.

## Introduction

The use of beta-adrenoceptor blockers (beta-blockers) as first line treatment for hypertension has declined in recent years due to inferior efficacy in the prevention of cardiovascular events when compared with vasodilatory antihypertensive agents [1–3]. One of the reasons for this is the lesser decrease of central than peripheral blood pressure (BP) during beta-blockade [4]. Still, beta-blockers remain the first-line therapy in patients with heart failure or recent myocardial infarction [5]. The pathophysiological mechanism for the inferior reduction in central BP with beta-blockers could be the longer ejection period during slower heart rate which allows the reflected wave to arrive during systole and therefore increase the systolic BP [6].

Augmentation index (AIx) is delineated as the proportion of the reflected pressure wave (augmentation pressure, AP) to pulse pressure (PP). It has been applied as a surrogate measure of arterial stiffness, but it is mainly a measure of wave reflections [7] that is also influenced by systemic vascular resistance (SVR) [8]. The position of AIx as an independent predictor of cardiovascular events is still controversial [9]. Taking into account the aforementioned pathophysiology it can be readily understood why a beneficial effect of beta-blockers on AIx has not been demonstrated [10, 11].

Amplification of PP is defined as the ratio of peripheral PP to central PP, and due to this phenomenon PP in peripheral arteries is higher than in central arteries [12]. Beta-blockers have been shown to decrease PP amplification [11], and this can be considered to reflect a reduction of central BP inferior to that of peripheral BP.

During orthostatic challenge, SVR and heart rate increase while cardiac output (CO) decreases [13]. In addition, AIx decreases in the upright position but remains constantly higher in hypertensive than normotensive subjects [14]. The wave reflections in the arterial tree are influenced by the time of the systolic ejection, heart rate, arterial stiffness, arterial branching, and SVR [8]. Beta-blocker therapy has profound effects on the regulation of heart rate and CO [11, 15]. In spite of the widespread use of these drugs, the effect of beta-blockers on central BP in the upright position remains unknown. Such information is very relevant, as significant proportion of the human daytime activity is performed in the upright position. To test the hypothesis whether the effects of beta-blocker treatment on central wave reflections and BP are accentuated in the upright position, we examined non-invasive hemodynamics in middle-aged men with never-treated grade I to grade II hypertension ingesting bisoprolol versus placebo in double-blinded randomized study.

## Methods

### Subjects

The study population of 16 non-smoking men with never-treated grade I to grade II essential hypertension was recruited via newspaper announcements (*n* = 14) and from occupational health care clinics (*n* = 2) [11]. The age range for possible inclusion in the study was 18–55 years, and the age range of the included subjects was 39–55 years. The definition of grade I to grade II hypertension was systolic office BP ranging from 140 to 180 mmHg, and diastolic BP ranging from 90 to 109 mmHg, according to the European evidence-based clinical guidelines [16].

All volunteers were interviewed and examined by a physician. Medical history, lifestyle variables, and status were documented, including BP measurements in the office (seated position, two brachial BP measurements using a sphygmomanometer, mean value recorded). Smoking was calculated as pack-years and smoking status was evaluated as current smoker, non-smoker, or ex-smoker. The time since smoking cessation in ex-smokers was recorded in years. Alcohol use was assessed as average ingestion of standard drinks (~12 g of absolute alcohol) during 1 week. The amount of exercise was the number of  ≥30 min exercise sessions per week that caused shortness of breath or sweating, as reported by the participants. Routine laboratory tests were taken [16]. A total of 24 subjects were examined and eight subjects were excluded on the basis of the following exclusion criteria: use of BP-lowering medication, secondary hypertension, BMI >35 kg/m^2^, current smoking, high consumption of alcohol (>24 restaurant doses/week), heart rate lower than 50 beats/min at physical examination, previous diagnosis of a heart disease, diabetes mellitus, disease of cerebrovascular or peripheral arteries, and pulmonary disorder.

Written informed consent of participation in the study was signed by all subjects. The ethics committee of Tampere University Hospital approved the study (investigation number R09103M) that conformed to the Declaration of Helsinki and was registered at ClinicalTrials.gov (NCT01742702).

### Study design and research drugs

**Fig. 1:**
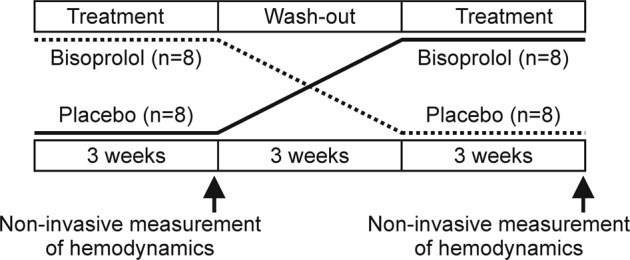
Study design. Sixteen male subjects were given bisoprolol or placebo for 3 weeks in a randomized, double-blind, cross-over study. A 3-week wash-out period took place between the treatment periods. Non-invasive hemodynamic measurements were carried out at the end of the treatment periods

This randomized, double-blinded cross-over study consisted of three 3-week periods (Fig. 1). The subjects were randomized into two groups that determined the order of the given treatments. The pharmacy of Tampere University Hospital, which was in no way involved in the examination of the study subjects, performed the randomization in blocks of four participants. During the first and third 3-week periods the subjects were given either bisoprolol or placebo once daily. The middle 3-week period was a wash-out phase so that no medications were given. The applied 5 mg daily dose of bisoprolol has been reported to significantly lower BP in Finnish men during a 24-h ambulatory BP registration [17]. The calculation of the sample size was based on the hypothesis that bisoprolol 5 mg daily will reduce diastolic BP ≥ 6 mmHg with a standard deviation (SD) of about 6 mmHg [18]. According to this calculation, ≥13 subjects were required to gain a statistical power of 90% (two-sided alpha = 0.05), and therefore altogether 16 subjects were recruited.

### Hemodynamic measurements

Simultaneous recordings using whole-body impedance cardiography and radial pulse wave analysis were performed at the end of the first and third 3-week periods in a research laboratory by a trained research nurse (Fig. 1) [13, 14]. Study subjects were asked to refrain from products containing caffeine, smoking, and heavy meals for ≥4 h, and from alcohol consumption for ≥24 h prior to the recordings. During ~10 min supine rest before the measurements, electrodes for impedance cardiography were placed on the body surface, a tonometric sensor for pulse wave analysis was placed on the left wrist over the radial artery, and a brachial BP cuff was attached to the right upper arm for BP calibration. The left arm with the tonometric sensor was abducted to 90° in a support, holding the wrist steady at the level of the heart during the measurements. Beat-to-beat hemodynamics were captured continuously for 10 min (5 min supine, 5 min upright).

### Pulse wave analysis and whole-body impedance cardiography

A tonometric sensor with automatic adjustment (Colin BP-508T®, Colin Medical Instruments Corp., USA) was applied to record radial BP and pulse wave form continuously, and the BP signal was calibrated at ~2.5 min intervals by use of the brachial cuff in the contralateral arm [13, 14]. The aortic pulse wave form was derived from the radial pulse wave using a validated generalized transfer function [19] by means of SphygmoCor® pulse wave monitoring (PWMx system, AtCor Medical, Australia). AIx (augmented pressure/PP*100%), AIx adjusted to heart rate 75/min (AIx@75), and Buckberg subendocardial viability ratio [20] were calculated from the aortic pulse wave with the SphygmoCor software.

Beat-to-beat heart rate, stroke volume, and CO was determined by the use of whole-body impedance cardiography (CircMon®, JR Medical Ltd., Tallinn, Estonia) [21]. To calculate SVR, the BP measurement from the radial tonometric sensor and CO by the CircMon® were used. SVR was normalized to the surface area of the body (SVR index, SVRI). With the recordings using CircMon®, stroke volume values correlate well with three-dimensional echocardiography (*r* = 0.781, bias 4.1 ml, 95% CI −2.2 to 10.4) [22], and CO measurements correlate well with values obtained by the thermodilution method (bias 0.00 l/min, 95% CI −0.26 to 0.26) and the direct oxygen Fick method (bias −0.32 l/min, 95% CI −0.69 to 0.05) [21]. Left cardiac work index (LCWI) values were calculated using the formula 0.0143*(mean aortic pressure−pulmonary artery occlusion pressure)*cardiac index, which was derived by the equation presented by Gorlin et al. [23]. Pulmonary artery occlusion pressure value was presumed normal (6 mmHg) and 0.0143 was the combined factor used to convert pressure from mmHg to cmH_2_O, volume to density of blood (kilograms per liter) and centimeters to meters.

### Laboratory analyses

Morning samples of blood and urine were obtained after ~12 h of fasting. The concentrations of sodium, potassium, creatinine, glucose, triglyceride, and total, high-density, and low-density lipoprotein (HDL and LDL, respectively) cholesterol in plasma were analyzed using Cobas Integra 700/800 (F. Hoffmann-Laroche Ltd., Basel, Switzerland) or Cobas6000, module c501 (Roche Diagnostics, Basel, Switzerland), and blood cell count by ADVIA 120 or 2120 (Bayer Health Care, Tarrytown, NY, USA). Urine dipstick analysis was performed using an automated refractometer (Siemens Clinitec Atlas or Advantus, Siemens Healthcare GmbH, Erlangen, Germany). GFR was calculated with the CKD-EPI creatinine-cystatin C—formula [24].

### Statistical analyses

Statistical analyses were performed using ANOVA for repeated measures to test differences between groups and interaction between time and group. Differences in changes of a variable were compared with paired samples *t*-test. *p*-Values < 0.05 were considered significant. The Shapiro–Wilk test was used to test that the hemodynamic variables were normally distributed and the equality of the variances was assessed with Levene’s test. In the figures the variables are depicted as means and standard error of means (SEM) for every minute of the 10 min recording period. The 3-week wash-out period between bisoprolol and placebo treatments was considered long enough to prevent any carryover effect on the placebo treatment phase, since the half-life of bisoprolol ranges from 10 to 12 h while the full treatment effect is attained within 2 weeks [25]. IBM SPSS statistics version 24.0 (Armonk, New York, USA) was used for statistical analyses.

## Results

### Study population

Every subject completed the study protocol, and their demographic data are presented as means and SDs in Table 1. The age range of the subjects was 39–55 years. There were no active smokers but seven subjects were ex-smokers with an average time from smoking cessation of 13 years [95% confidence intervals (CI) 7.7, 18.7]. Average alcohol consumption (self-reported) was 5.2 standard doses [2.7, 7.8] (each containing 12 g of alcohol) and frequency of physical exercise sessions 2.7 times per week [1.9, 3.4] (one session ≥ 30 min). According to Cornell’s voltage criteria (RaVL + SV3 > 28 mm) two of the subjects had left ventricular hypertrophy. Urine dipstick analyses showed no proteinuria or hematuria while average blood cell counts, and concentrations of sodium, potassium, and fasting plasma glucose were within the normal reference values. One subject had a small elevation in plasma creatinine concentration (113 µmol/L, corresponding estimated glomerular filtration rate 70 mL/min/1.73 m^2^). The ranges of plasma lipid values were as follows: total cholesterol 3.7–6.4 mmol/L, LDL cholesterol 2.1–4.5 mmol/L, HDL cholesterol 0.9–2.0 mmol/L, and triglycerides 0.52–2.4 mmol/L (Table 1).

**Table 1:** Demographic and laboratory data (*n* = 16)

Variable	Mean	SD
Age, years	48.4	5.5
Weight, kg	92.5	11.7
Height, cm	180	5.8
Body mass index, kg/m²	28.6	3.4
Waist circumference, cm	103	9.3
*Office BP at screening*		
Systolic BP, mmHg	149	9.6
Diastolic BP, mmHg	99	4.4
Heart rate, beats/min	67	10.8
*Laboratory values*		
Hemoglobin, g/L	153	8.8
Potassium, mmol/L	3.9	0.3
Sodium, mmol/L	141	1.5
Creatinine, µmol/L	82	12.6
Cystatin-C, mg/L	0.89	0.09
Calcium (total), mmol/L	2.33	0.13
Total cholesterol, mmol/L	5.1	0.8
HDL-cholesterol, mmol/L	1.3	0.3
LDL-cholesterol, mmol/L	3.2	0.8
Triglycerides, mmol/L	1.4	0.6
Fasting plasma glucose, mmol/L	5.6	0.4
Estimated GFR, ml/min/1.73 m^2^	95.8	10.7

*BP* blood pressure, *GFR* glomerular filtration rate estimated using the CKD-EPI-Creatinine-cystatin-C formula [23]

The medications in regular use by the study participants were statin therapy for hypercholesterolemia (*n* = 2), selective serotonin reuptake inhibitors for depression (*n* = 2), and thyroxine treatment for hypothyroidism with a steady euthyroid state (*n* = 1). In addition, gastro-esophageal reflux had been diagnosed in two subjects, but they did not need any regular medications.

### Hemodynamic measurements

**Fig. 2:**
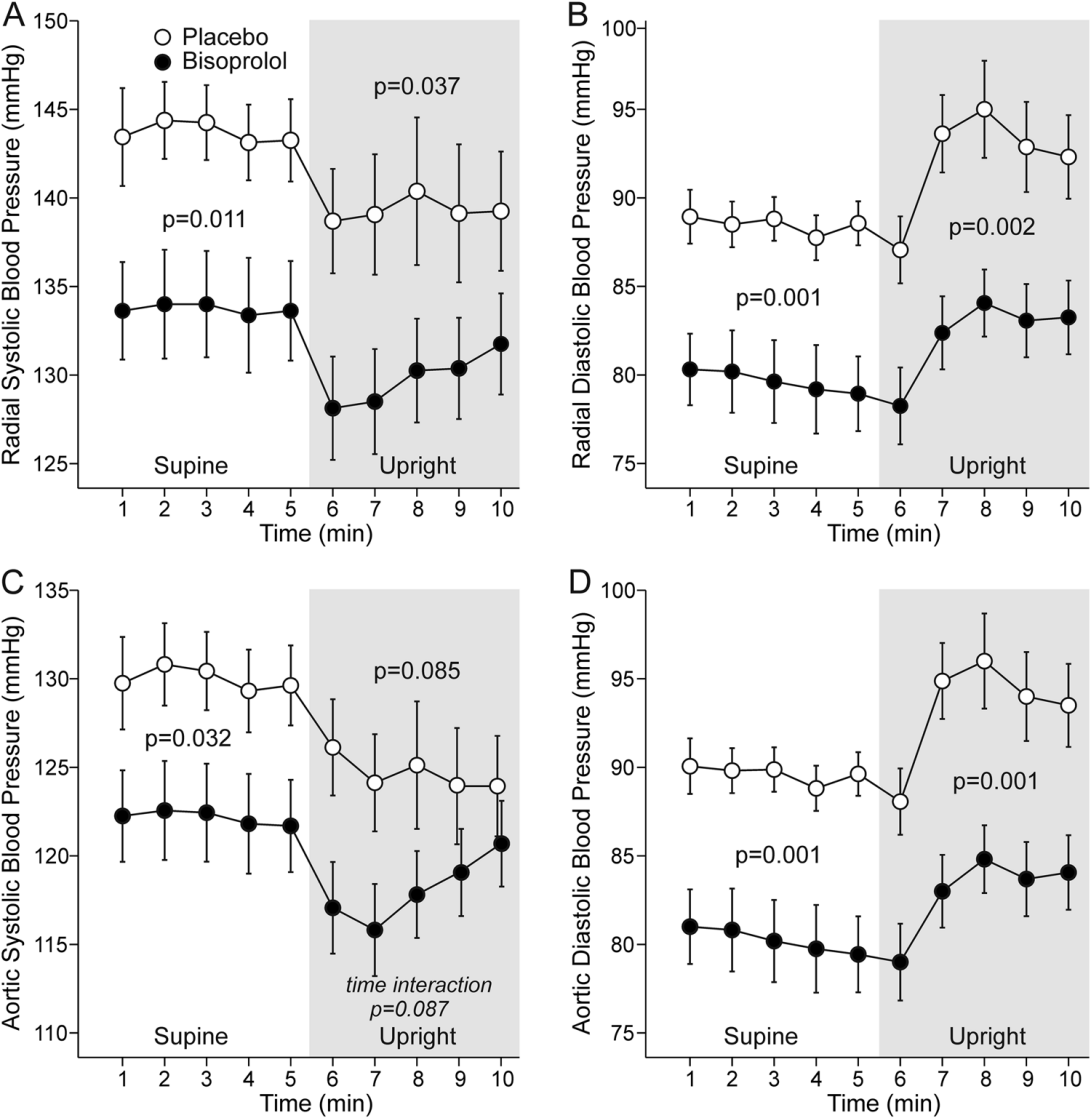
Radial systolic (**a**), radial diastolic (**b**), aortic systolic (**c**), and aortic diastolic (**d**) blood pressure during treatment with bisoprolol or placebo. Variables are depicted as mean ± SEM for every minute of recording. *p*-Values represent ANOVA for repeated measures between bisoprolol and placebo during supine (5 min) and upright (5 min) positions. Time interaction is calculated from the whole 10 min recording period

In the supine position radial systolic BP was 10 mmHg [4.8, 15.1] and diastolic BP 9 mmHg [3.8, 13.9] lower, and aortic systolic BP 8 mmHg [2.7, 12.9] and diastolic BP 9 mmHg [4.3, 14.5] lower during bisoprolol than during placebo. In the upright position bisoprolol treatment reduced radial systolic BP by 10 mmHg [4.0, 15.0], diastolic BP by 10 mmHg [6.2,13.8], and aortic diastolic by 10 mmHg [6.6, 14.1] (Fig. 2a–d), but contrary to the supine position, aortic systolic BP was not significantly reduced (*p* = 0.085).

**Fig. 3:**
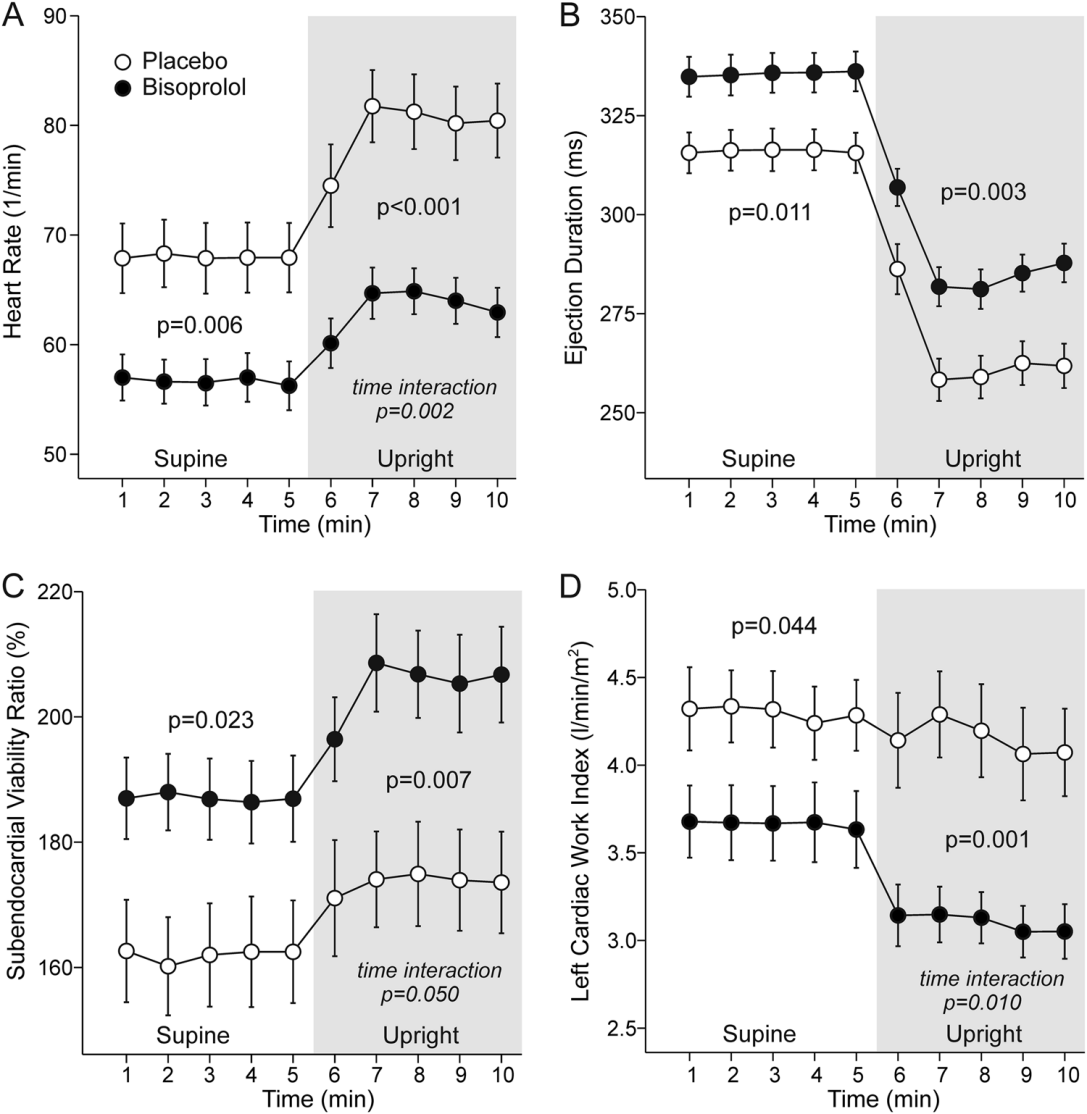
Heart rate (**a**), ejection duration (**b**), subendocardial viability ratio (**c**), and left cardiac work index (**d**) during treatment with bisoprolol or placebo. Mean ± SEM, calculations as in Fig. 2

Bisoprolol reduced heart rate in both supine (−11 beats/min [−7.9, −14,7]) and upright (−16 beats/min [−12, −20]) positions (Fig. 3a), while the decrease in heart rate was significantly greater upright than supine (−5 beats/min [2.8, 7.4], *p* < 0.001). Ejection duration was prolonged in supine (+20 ms [12.4, 26.7]) and upright (+23 ms [15.6, 30.4]) positions with bisoprolol (Fig. 3b). SEVR increased both supine (+25 percentage points [14.0, 36.1]) and upright (+31 percentage points [21.5, 41.0]), while LCWI was reduced in supine (−0.7 kg*m/m^2^ [−0.26, −1.1]) and upright (−1 kg*m/m^2^ [−0.65,−1.1]) positions with bisoprolol when compared with placebo (Fig. 3c, d).

**Fig. 4:**
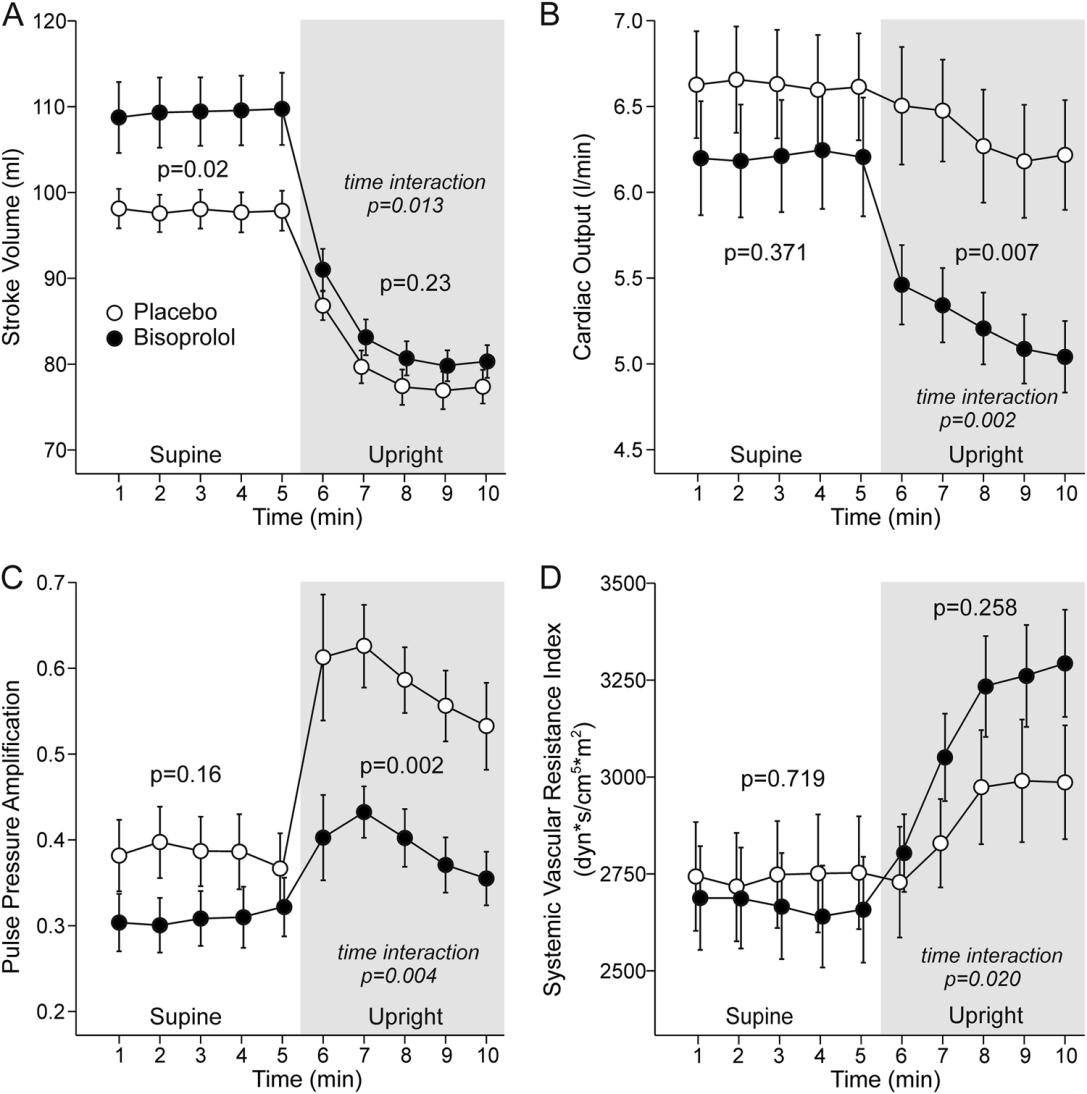
Stroke volume (**a**), cardiac output (**b**), pulse pressure amplification (**c**), and systemic vascular resistance (**d**) during treatment with bisoprolol or placebo. Mean ± SEM, calculations as in Fig. 2

Stroke volume was significantly higher with bisoprolol in the supine position (+12 [5.5, 17.5] ml) but not during the head-up tilt (Fig. 4a), whereas CO was not changed in the supine position but was significantly lower during the upright position (−1.1 L/min [−0.7, −1.5], Fig. 4b). PP amplification was not changed with bisoprolol in the supine position, but was reduced (−19 percentage points [−13, −25]) in the upright position (Fig. 4c). When analyzed using ANOVA for repeated measures, neither supine nor upright SVRI was significantly different from placebo during bisoprolol. However, a significant time interaction was observed, and the upright increase in SVRI was 2.7-fold higher after bisoprolol when compared with placebo (634 vs. 233 dyn*s/cm^5^*m^2^, *p* = 0.003) (Fig. 4d).

**Fig. 5:**
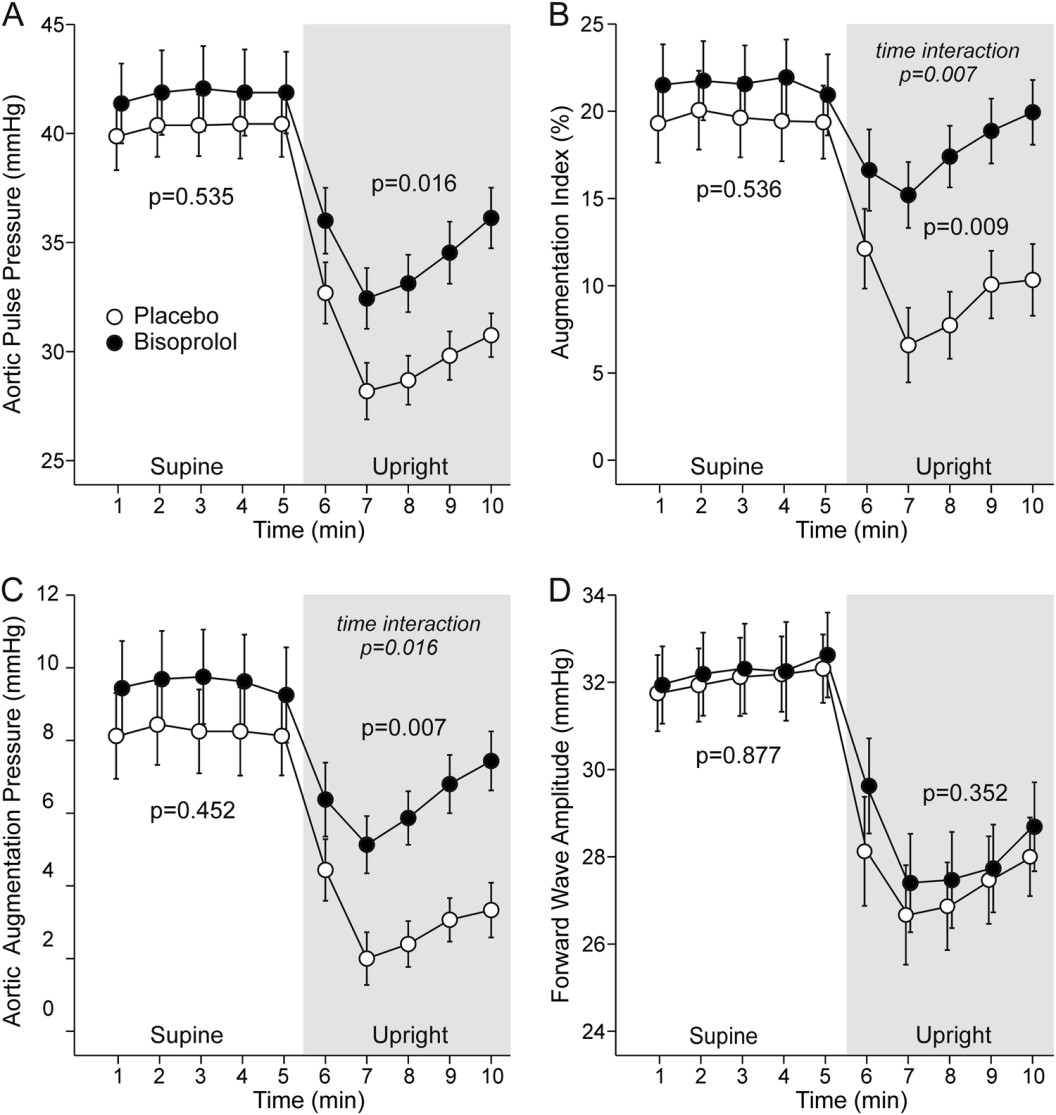
Aortic pulse pressure (**a**), augmentation index (**b**), aortic augmentation pressure (**c**), and forward wave amplitude (**d**) during treatment with bisoprolol or placebo. Mean ± SEM, calculations as in Fig. 2

While there were no changes in aortic PP or AP in supine measurements, upright aortic PP was increased+4 mmHg [1.7, 7.1] and aortic AP +3 mmHg [1.9, 4.3] with bisoprolol (Fig. 5a, c). AIx remained unchanged in supine position, but was significantly higher (+7.8 percentage points [4.8, 10.6]) upright with bisoprolol than with placebo Fig. 5b). The heart rate-related AIx@75 was not changed during bisoprolol treatment either supine (*p* = 0.368) or upright (*p* = 0.418) (not shown). No difference in forward wave amplitude between the bisoprolol and placebo treatments was found (Fig. 5d). Radial PP was unchanged during bisoprolol both supine (*p* = 0.6) and upright (*p* = 0.8) (not shown).

## Discussion

In this randomized, placebo-controlled double-blind cross-over study, non-invasive evaluation of hemodynamics was carried out in men with mild to moderate primary hypertension during non-vasodilating beta-blocker therapy. The results show that bisoprolol did not lower central systolic BP in the upright position although it was efficient in reducing peripheral BP. Moreover, the harmful influences of bisoprolol on central PP and wave reflection were uncovered in the upright position. This emphasizes the influence of body posture on the hemodynamic changes induced by beta-blocker therapy. The supine hemodynamics and home BP measurements of the present study subjects were previously reported [11], and the observed reductions in heart rate and BP correlate well with former results concerning bisoprolol treatment [17], indicating good adherence to the medication.

Although the position of beta-blockers in the treatment of essential hypertension has changed, they remain a mainstay of pharmacotherapy after myocardial infarction. We observed a decrease in LCWI, indicating reduced workload of the left ventricle during bisoprolol therapy, and an increase in SEVR that reflects an improved ratio between myocardial oxygen supply and demand. These hemodynamic changes can explain the antianginal effect of beta-blockers.

During orthostatic challenge, blood pools in the lower extremities resulting in a decrease in venous return to the heart. As compensatory mechanisms SVR and heart rate increase to maintain the level of BP. Despite this adaptation, CO usually decreases, but there is significant inter-individual variation in the magnitude of these changes [26]. Of note, the phenotype of the hemodynamic response to upright posture seems rather persistent over time [27]. Heart rate reduction is a typical effect of non-vasodilating beta-blockers and we found that the reduction was more pronounced in the upright than in the supine position. Upright CO was also reduced by bisoprolol, and this can be attributed to the reduction of heart rate since no change in stroke volume was observed (CO = stroke volume × heart rate). Subsequently, pharmacological inhibition of the increase in heart rate during orthostatic challenge altered the regulation of SVR, and this variable showed an almost threefold increase in the upright position when compared with the response during placebo.

AIx was clearly increased during bisoprolol in the upright position. The level of AIx is inversely related with heart rate [28] and directly with SVR [8]. Therefore, the observed changes in these hemodynamic determinants of wave reflection provide the likely explanation for the upright increase in AIx during bisoprolol treatment. Furthermore, the increase in aortic PP can be attributed to enhanced wave reflection, as there was no change in the upright forward wave amplitude during bisoprolol. In addition, enhanced wave reflection also explains the reduced PP amplification in the upright position during bisoprolol.

Previous studies evaluating the effect of upright posture on wave reflection have suggested that the associated increase in heart rate may not fully explain the observed reduction in AIx in non-medicated healthy subjects [13, 29], and decreased stroke volume could partially explain why AIx is lower upright than supine. In the present study, no difference in upright stroke volume was observed between the bisoprolol and placebo treatments. In addition, the heart-rate-related variable AIx@75 did not differ during bisoprolol versus placebo, which emphasizes the significance of heart rate reduction in the observed increase in wave reflection during beta-blockade. An experimental study using simulated arterial tree proposed that the dependency of wave reflection on heart rate is influenced by arterial viscoelasticity [30]. In the study reported here such changes in arterial viscoelasticity that would only have influenced upright hemodynamics seem unlikely. Aortic reservoir pressure has also been postulated as a variable that influences the magnitude of the Aix [31–33]. However, the reservoir pressure concept is not without controversy as the model has been claimed to diminish the reflected waves, create artefactual waves [34], and even to be based on incorrect physiological concepts [35].

Pulse wave velocity (PWV) is an important determinant of AIx since the kinetics of the reflected wave depend on the traveling speed of the pressure wave in the arterial tree [7]. In our previous report focused on the supine hemodynamics after bisoprolol, PWV was decreased most likely due to the parallel reduction in BP [11]. Such a change would favor a reduction, not an increase, in the level of the AIx. We did not address PWV during the head-up-tilt, but previous reports have shown an increase in PWV with upright posture [36], possibly due to decreased arterial compliance during the changes in hydrostatic pressure induced by orthostatic challenge.

The mechanism behind the beta-blockers’ inferior reduction of cardiovascular events when compared with vasodilatory antihypertensive drugs is still not completely understood [1–3]. The CAFÉ-trial showed that despite similar BP reduction at the brachial level with amlodipine-based and atenolol-based therapy [4], the BP and PP lowering effect at the aortic level was more pronounced with amlodipine. Higher AIx during atenolol was attributed to the longer ejection period during slower heart rate, allowing the peak of the reflected pressure wave to augment the central systolic pressure. This probably has clinical relevance since central PP has been shown to predict cardiovascular outcomes better than peripheral PP [37], while this variable was also associated with cardiovascular endpoints in a post-hoc analysis of the CAFE trial [4]. Importantly, previous studies about the hemodynamic effects of beta-blockers have been exclusively carried out in the supine position and have rarely included comprehensive evaluation of central BP and its determinants. To our knowledge, the finding that upright posture accentuates the beta-blockers’ deleterious effects on central BP has not been shown before. Of note, the incidence of ischemic stroke is highest during morning hours [38], a moment after waking up and probably after changes in body posture. The hemodynamic alterations in response to the changes in body posture might contribute to this circadian variation and calls for further study in subjects on beta-blocker therapy.

There are some limitations in this study. The number of subjects does not allow multivariate analyses to be carried out. However, the findings were very clear in this population. The investigations were done in men only so the results might not be directly applicable to women. Mathematical transfer functions were required to derive central BP and other hemodynamic variables but good correlation with direct measurements using these methods has been shown [22].

Bisoprolol decreased peripheral and central BP in men with grade I to grade II hypertension. However, central systolic BP was not reduced during head-up tilt. In addition, the non-beneficial effects on central BP and wave reflection with bisoprolol were especially manifested in the upright position. These findings show that central BP regulation is substantially affected by posture during treatment with beta-blockers. Altogether, the influence of posture should be taken into account when evaluating the effects of antihypertensive agents on hemodynamics.

## Summary

### What is known about topic

- Beta-blockers do not prevent cardiovascular events to same extent as other antihypertensive drugs.
- Beta-blockers do not reduce central blood pressure as well as vasodilatory antihypertensive drugs despite similar reduction of peripheral blood pressure.

### What this study adds

- In supine measurements bisoprolol reduced peripheral and central blood pressure in never-treated grade I to grade II hypertensive men with no effect on central wave reflection or central pulse pressure.
- During passive upright tilt, the effect of bisoprolol on central pulse pressure and wave reflection was clearly detrimental and there was no reduction in central systolic pressure compared to placebo.
- Hemodynamic measurements performed at rest seem to underestimate the effect of beta-blocker therapy on the regulation of hemodynamics as harmful influences of bisoprolol on central blood pressure and wave reflection were manifested only during upright tilt.

## Supplementary information

Letter from JHH editorial office
