# Supplementary material for: Adverse influence of bisoprolol on central blood pressure in the upright position: a double-blind placebo-controlled cross-over study
Source: J Hum Hypertens. 2019 Mar 18;34(4):301–10. doi: 10.1038/s41371-019-0188-9 (PMC7165126; doi:10.1038/s41371-019-0188-9)
Supplement: Supplementary file 1 — Letter from JHH editorial office [file 41371_2019_188_MOESM1_ESM.pdf]

From: Virginia Mercer
Sent: Thursday 16 August 2018
Subject: JHH Call for Quality Research
Recipient: Antti Tikkakoski

Dear AJ Tikkakoski,

On behalf of the Editor-in-Chief, Prof Michael Stowasser, of the *Journal of Human Hypertension* **,** I would like to congratulate you on the success of your 2013 Article, *“Hemodynamic alterations in hypertensive patients at rest and during passive head-up tilt”* published inJournal of Hypertension. We at *JHH* notice and value exceptional research and encourage you to send your next research or review paper to *JHH*.

JHH is an international publication with an impact factor score of **2.433**. We believe that our patients benefit from robust scientific data that are based on well conducted clinical trials. We also believe that basic sciences are the foundations on which we build our knowledge of clinical conditions and their management. Towards this end, although we are primarily a clinical based journal, we also welcome suitable basic science studies that promote the understanding of human hypertension. The journal aims to perform the dual role of increasing knowledge in the field of high blood pressure as well as improving the standard of care of patients. The editors will consider for publication all suitable papers dealing directly or indirectly with clinical aspects of hypertension, including but not limited to epidemiology, pathophysiology, therapeutics and basic sciences involving human subjects or tissues. We also consider papers from all specialties such as ophthalmology, cardiology, nephrology, obstetrics and stroke medicine that deal with the various aspects of hypertension and its complications.
Points of interest to consider:

· *JHH* has a large readership (over 500,000 views per year) and its content is accessed and read globally due to it being hosted on nature.com
· *JHH* ensures a quick turnaround time for authors
· *JHH* authors can freely share a read-only, full text version of their paper through SharedIt. This link can be shared with colleagues and friends, as well as being posted on social media platforms, institutional repositories and author websites
· *Journal of Human Hypertension* Young Investigator Award – an opportunity to celebrate and recognize exceptional research outcomes in the field of human hypertension.
· See *JHH*’s new web collection on Energy Expenditure and Body Composition and keep your eyes peeled for another on Pregnancy, Growth and Childhood Disease publishing at the end of the month.
· *JHH* has a new, user-friendly, easy to navigate website, that promotes immersive reading
· *JHH* content is included in Springer Nature cross company campaigns, such as the recent DNA Day campaign, as well as the ground breaking Change the World campaign and Nature Outlooks, which tackle topics of scientific, clinical and societal interest.
· *JHH* has a Twitter account to actively encage with our readers and share the latest research from the journal

The Editorial Office would be happy to respond to any questions you might have regarding the submission process. Please refer to this letter in your submission or in email to the editorial office upon submission. **We would be delighted to waive the colour figure charges for your next submission.**

We invite you to review our Guide for Authors and also visit our Author Tutorials site, an invaluable resource which provides helpful tips, guides and quizzes to assist you in the writing and submission of your paper.

Thank you for your support of *JHH*, and we hope to receive your next paper and repeat your success.

Best wishes

Virginia Mercer
Publishing Manager

On behalf of
Prof Michael Stowasser
Editor-in-Chief, *Journal of Human Hypertension*
